# Omega 3 in Childhood Migraines: a Double Blind Randomized Clinical Trial

**Published:** 2016

**Authors:** Afshin FAYYAZI, Ali KHAJEH, Ahad GHAZAVI, Mahsha SANGESTANI

**Affiliations:** 1 Department of Pediatrics, Hamedan University of Medical Sciences, Hamedan, Iran; 2 Department of Pediatrics, Children and Adolescent Health Research Center, Zahedan University of Medical Sciences, Zahedan, Iran; 3 Department of Pediatric Neurology, Urmia University of Medical Sciences, Urmia, Iran; 4 Hamedan University of Medical Sciences, Iran

**Keywords:** Children Migraine, Prevention, Treatment, Omega 3, Fish oil

## Abstract

**Objective:**

The effect of using omega-3 to prevent migraine attacks has been raised in recent studies. The majority of these studies have been conducted in adults. Conversely, other studies have yet to confirm the effect of omega-3. The main purpose of this study was to assess the effects of omega-3 in the prevention of migraine attacks in children.

**Materials & Methods:**

In this study, children aged 5–15 years with a diagnosis of migraine were randomly assigned to case and control groups. The case group was treated with sodium valproate and 1 g of omega-3; the control group was treated with sodium valproate and a placebo for 2 months. The severity of attacks was evaluated before and after the treatment using PedMIDAS and parental satisfaction (CGI) using a 7-point Likert scale.

**Results:**

In this study, 12 cases and 13 controls were enrolled. The average number of headache attacks per month decreased significantly in both groups after starting the treatment but there was no significant difference between the two groups. The severity of attacks decreased significantly in both groups after starting the treatment but it was not significant between them. Examination of the CGI average showed the average was 6.08 (SD = 0.52) in the case group and 6.07 (SD = 0.65) in the control group.

**Conclusion:**

The present study indicated that omega-3 with a dose of 1 mg per day has no effect in reducing the severity and frequency of migraine attacks in children. Sodium valproate was effective in reducing the frequency and severity of attacks.

## Introduction

Migraines are a common disorder in children and observed in 10.6% of children aged 5–15 years (1). The mean age of onset of migraine symptoms is 7 year of age in boys and 10.9 years of age in girls (2). Frequent migraine attacks may cause concern for parents in addition to their impact on the quality of life of children (3). Migraine prevalence has increased up to 3 times over the past three decades (4). The exact cause of this issue is unknown. However, increased environmental stress and changes in diet (increased consumption of caffeine and aspartame in beverages) could be the reasons for the increase (4). It is estimated that the average absence from school for children with migraines is 1.5 weeks higher than for peers (5). If the patients suffer from frequent headache attacks or with severe disabilities, they need to have daily preventive medication (3). Prophylactic treatment is an important part of migraine treatment and its goal is to reduce the frequency, severity, and duration of headaches as well as to improve the effect of medications to stop the headaches (6). These therapeutic measures include a variety of items such as pharmacological and nonpharmacological methods, dietary supplements, and herbal ingredients (6). Conventional medications for children, which their preventive impact on migraine has been proven in clinical trial studies, include items such as sodium valproate, topiramate, propranolol, amitriptyline, naproxen, and flunarizine (7). Nonpharmacological approaches focus on improving the quality of sleep, reducing daily stress, exercise, and modifying dietary patterns (6). The methods based on the dietary supplements and natural ingredients focus on the effects of L-carnitine, vitamin B-2, fish oil, omega-3, and magnesium in preventing migraines (8)(9)(10)(11). The use of omega-3 polyunsaturated fatty acids in the prevention or treatment of migraine has received much attention in recent studies (10) (11)(12). Omega-3 interferes with the conversion of omega-6 to eicosanoids; therefore, reduces the production of prostaglandins and leukotrienes (11). The hypothesis of perivascular inflammation and neurogenic inflammation in incidences of migraines may explain the anti-inflammatory effects of omega-3 to reduce migraines (11). The investigations carried out in this area were mostly in adults and with different results (10) (11) (12). The main purpose of this study was to compare the effects of omega-3 as an adjuvant in reducing the severity of migraine attacks in children.

## Materials & Methods

In this study, children aged 5–14 years of age who were referred to the Pediatric Neurology Clinic of Besat Hospital and Imam Khomeini Clinic of Hamedan with complaints of headache and were diagnosed with a migraine according to the International Headache Society Revised Criteria for Migraine in Children as well as with frequent attacks (more than one attack per week or more than three attacks per month or more than one day leaving school per month) were included. The Ethics Committee of the Hamadan University of Medical Sciences approved our study. Patients were divided into two groups of at least 12 individuals and matched for age (children aged 5–14 years of age) by a simple random sampling method using a random number table. The first group (control group) was treated with sodium valproate syrup or tablet with a dose of 20 mg per kg of body weight (maximum 200 mg twice daily) in two divided doses daily in combination with a placebo capsule. The second group (case group) was treated with sodium valproate tablet or syrup with a dose of 20 mg per kg of body weight (maximum 200 mg twice daily) in two divided doses daily in combination with an omega-3 capsule (Zahravi pharmaceutical company) containing 1 g of fish oil (180 mg eicosapentaenoic acid (EPA) and 120 mg docosahexaenoic acid (DHA)). Placebo capsules were prepared by Zahravi pharmaceutical company with a shape, color, and packaging quite similar to omega-3 capsules and without any effective substances. After selecting the patients for inclusion into the project and offering explanations to the parents and to obtain informed consent, patients were randomly assigned to one of the two groups (A or B) and a code was assigned to them. Then, without informing the patients and the physician about the medication content of group A or B, medications were given to the patients by the physician and they were treated and followed for at least 2 months and the severity and frequency of headaches were recorded. The codes were unveiled after completion of the treatment duration and recording of patient information. In some patients, a brain CT scan and an MRI were conducted before starting the study. Moreover, these imaging measures were carried out based on the need in case of a change in the clinical symptoms during the study such that it was not justifiable with a diagnosis of migraine. If the diagnosis was changed, the person was excluded and replaced by another suitable for the study. Monthly assessments of patients were performed. Routine monitoring was performed for sodium valproate intake. Other appropriate medications were prescribed in cases of the exacerbation of symptoms and no response to treatment. During the attacks, treatments were used to stop headaches if needed. The demographic data including age, gender, and information about the history and symptoms were recorded before starting treatment. The number of headache attacks per month before and after the treatment was recorded by questioning the parents. The severity of headaches before and after the treatment was evaluated according to the Pediatric Migraine Disability Assessment Score (PedMIDAS). The headache severity was divided into grade 1 (0–10), grade 2 (11–30), grade 3 (31–50), and grade 4 (greater than 50) according to the PedMIDAS questionnaire. Grade 1 had the lowest and grade 4 had the highest headache severity. Furthermore, Clinical General Impression (CGI) was evaluated using a 7-point Likert scale. Grade 1 is very dissatisfied and grade 7 is quite satisfied. Patients were revisited every two weeks for 2 months after the treatment and the severity of migraine attacks after the treatment were evaluated. The outcome of the treatment in the two groups was analyzed using SPSS (ver 16) as the impact on the number of headache attacks and headache severity in each group. This study was registered as an Iranian clinical trials with registration number IRCT2013092914809N1.

## Results

In the present study, 25 patients from the studied population were included in the project. The case group involved 12 patients who were treated with omega-3 capsules and sodium valproate; and the control group included 13 patients who were treated with sodium valproate and a placebo. The mean age of patients in this study was 10.36 years (SD = 2.88). Gender analysis of the studied patients showed that of the 25 patients, 11 patients were male (44%) and 14 were female (56%). The results of the demographic data showed no significant differences between the two groups. The mean number of headache attacks per month before starting the treatment showed that the average in the case group was 13.75 attacks per month (SD = 8.36); and in the control group, this average was 16.30 attacks per month (SD = 10.38) (PV = 0.507). An assessment of the mean number of headache attacks per month after the treatment showed that in the case group the average was 2.91 attacks per month (SD = 2.91) and in the control group, the average was 3.15 attacks per month (SD = 3.15) (PV = 0.822). The number of attacks before and after the treatment decreased significantly in both groups (placebo: P = 0.000 and omega-3: P = 0.000). Evaluation of headache severity using PedMIDAS (grades 1–4) before the treatment showed that in the case group the average of this scale was 2.08 (SD = 1.08); and in the control group, the average of this scale was 2 (SD = 0.92) (PV = 0.837). An evaluation of headache severity using PedMIDAS (grades 1–4) after the treatment showed that in the case group, the average of this scale was 1.08 (SD = 0.289) and in the control group, the average of this scale was 1 (SD = 0.00) (PV = 0.308). The severity of symptoms before and after the treatment decreased significantly in both groups (placebo: P = 0.002 and omega-3: P = 0.004). Assessment of the average of CGI (1–7) showed that in the case group the average was 6.08 (SD = 0.52); and in the control group, the average was 6.07 (SD = 0.65) (PV = 0.978). As regards the side effects of omega-3, there was only one case of nausea after omega-3 intake where the patient was not willing to continue the treatment and the patient was excluded from the study. No other side effects were observed in the other patients.

## Discussion

Previous studies have been conducted recently on the effects of omega-3 in neurological diseases such as autism, dementia, depression, hyperactivity, and headaches (10) (13) (14) (15). Bloch et al. has demonstrated a small but significant benefit of omega-3 fatty acid supplementation in improving ADHD symptoms in a systematic review and meta-analysis study (16). Meta-regression also demonstrated a significant association between EPA dose within supplements (80–750 mg) and supplement efficacy. In this study, we tried to determine which omega-3 is beneficial in treating child migraines. It was determined that omega-3 has no effect on reducing the frequency and severity of migraine attacks in children. However, the outcome of treatment with sodium valproate showed that it was significantly effective in both groups. Tajmirriahi et al. investigated the effects of fish oil (1 g omega-3 capsule) in combination with sodium valproate compared to placebo and sodium valproate in 68 adult patients. Patient headache severity (by a 10-point visual analog scale, VAS), frequency of headache per month, and duration of headache were determined before and after 1, 2, and 3 months of treatment. There was a significant reduction in headache frequency (P = 0.044) and headache severity (P = 0.046) in the case group after the first month (10). Harel et al. compared the prophylactic effects of omega-3 with a placebo (olive oil) on 23 patients with a history of migraines that led to positive results and omega-3 oil significantly reduced the frequency of migraine attacks (11). Pradelier et al. indicated from the daily intake of 6 g of omega-3 versus placebo for 4 months did not show positive results in the management of migraines. In this study, 198 patients with a history of recurrent migraine were first treated with omega-3 oil and then discontinued the drugs and again, one month later they were treated with a placebo. There were no significant differences between the two treatments in terms of reducing the frequency of headaches (12). Different patient’s age and evaluation methods are probably the main reasons for the different result in our study.

**In conclusion**, although omega-3 was less effective than a placebo in our study, it seems that conducting other studies with larger sample sizes and if possible without the presence of other preventive medications may lead to different results.
